# How smart senior care can achieve value co-creation: Evidence from China

**DOI:** 10.3389/fpubh.2022.973439

**Published:** 2022-09-15

**Authors:** Liping Fu, Tong Pei, Jie Yang, Jiarui Han

**Affiliations:** ^1^Center for Social Science Survey and Data, College of Management and Economics, Tianjin University, Tianjin, China; ^2^College of Politics and Public Administration, Qinghai Minzu University, Xining, China; ^3^School of Public Administration, Hainan University, Haikou, China

**Keywords:** smart senior care, value co-creation, practical logic, driving factor, grounded theory, digital divide

## Abstract

With the rapid rise of artificial intelligence, smart senior care has become a new trend for future development. The collection of “Typical Cases of Chinese Elderly Service Industry Development” is selected by the script materials. The main purpose of this article is to investigate how smart senior care can achieve value co-creation by grounded theory. This study explores the phenomenon of value co-creation in the participation of multiple actors in smart senior care services. Findings show that institutional guarantee, technical intake, market empowerment, emotional support, service interaction, and reciprocity norm are identified as the driving factors for value co-creation. In addition, the behavioral processes of value co-creation include multi-actor value consensus, co-creation environment establishment, practical value co-creation, public value sharing, and diffusion of service added value in smart senior care. Finally, this study constructs a practical logic model of achieving value co-creation. It extends and enriches the scope of the value co-creation theory. This study confirms that value co-creation can be effectively achieved in smart senior care by the above-mentioned ways, revealing its driving factors and behavioral processes. The article expands on the application of value co-creation in the field of public healthcare. The results have important theoretical and practical significance for narrowing the public service equalization gap.

## Introduction

According to the 7th China Census, there are 264 million people aged 60 and above, accounting for 18.7% of the total population in China. Conventional senior care can no longer meet the elderly basic requirements (1). Following the fast growth of new-generation information technology, smart senior care (SSC) is gradually becoming a new development direction to alleviate this dilemma, which is first proposed by the British Life Trust (2). SSC is an expansion of the conventional aged healthcare service that incorporates modern technology to enhance the living circumstances of the elderly (3). Information and communication technologies (ICTs) have promoted the continuous upgrading of medical service modes, making it easier to obtain medical resources on the Internet, and providing a new method for the public to seek health information and promote healthy communication (4, 5). However, disruptive and fast-expanding ICTs, such as robots, big data, and telehealth, make healthcare services increasingly complex. In this context, the elderly group is generally less receptive to ICTs. This reluctance is a crucial challenge for service innovators in SSC and other technology areas. In order to improve the service acceptability of innovation, service providers may transform technology-based services into value for a wide range of consumers.

Existing research is principally analyzed and presented from a practical level for the design of the SSC platform and its key technological concerns. Studies have developed a home monitoring system for the elderly living alone based on the ZigBee protocol (6). Such a system can collect smart home usage and physiological data like heart rate and skin temperature from the elderly to monitor their health status. Other studies could try to set up internal sensor data cluster systems, environmental monitoring systems, assisted living systems, and remote care detection systems to track tegular life (7). During the period of COVID-19, the importance of such digital health became more prominent (8). Although the range of SSC devices has made geriatric health monitoring more convenient than before, privacy concerns may be tough to overlook. In other words, people's willingness to participate in health care through ICTs is primarily influenced by privacy breaches (9). Previous studies, on the other hand, might provide several clues about how privacy considerations interact with ICTs to impact user participation in value co-creation (10).

In reality, SSC can use modern information technology to fill in the shortcomings of the family senior care function (11). Internet technology connects general hospital professionals with patients in the Web Cloud, enabling them to consult with a doctor from their own homes. Furthermore, SSC may also provide children who are away from home the chance to learn about their parents' health. For example, the elderly use a variety of medical equipment, and then their children may use the Internet to learn about their heart rate, blood pressure, and other physical information of parents. As a part of social support for the elderly, intergenerational support is vital to the physical and mental health of the parents (12). Additionally, SSC may also enhance the quality of life of the elderly by altering the way they are cared for. As interaction is required (13). In other words, it is critical to determine the impact of various interactions between service providers and older adults on the value co-creation process and how to design effective quality care processes.

As an innovative approach to creating value, value co-creation has attracted the academic community's interest (14). Since its inception, the value co-creation theory has been chiefly debated in business services. Vargo and Lusch tried to extend its scope of application to the field of public services (15). Afterward, value co-creation in healthcare services has also received attention (16). According to research, value co-creation is appropriate for exploration in knowledge-intensive service areas (17, 18). Service-Dominant (SD) logic considers the participation of multiple actors are a fundamental part of the value co-creation process (19). Specifically, value co-creation allows multiple actors to create value through interaction. Then such interaction helps customers become effective co-creators to obtain better service outcomes (17). Scholars also attempt to extend the application of SD logic to public services and claim that value co-creation theory could be applied to government-provided public products and services (20). According to previous research, establishing a co-creation environment is essential for achieving government-public interaction and value co-creation (21). Therefore, the participating actors are more diverse, and the co-creation process is more complex. One of the key points is how various social actors participate in the value co-creation of SSC services.

Value co-creation can be achieved directly through interactions between patients and healthcare providers in SSC. Specifically, ICTs are generally used as an intermediary to integrate various resources and achieve indirect interaction in SSC. For example, mobile technology makes it easier for patients to participate in the co-creation process (22). ICTs might enhance collaborative interactions between patients and medical institutions to facilitate the achievement of value co-creation. Medical infrastructure such as the Internet of Things (IoTs), Medical Cloud, Mobile Internet, and Wearable Devices are already integrated into SSC, which supports smart decision-making. The IoTs and wearable technologies might be quite popular and crucial in SSC (23). However, Chinese seniors have a limited understanding of the use of these technologies, resulting in a considerable mismatch between technology use and actual demand (24). This makes the elderly unable to fully enjoy the convenience brought by smart services and eventually become “digital refugees” (25). In response, other countries have adopted several initiatives and policy interventions to promote access and utilization of health care services in older adults (26, 27). Therefore, the importance and urgency of digital divide governance are becoming more and more prominent in China.

Value co-creation has previously been separated into four stages: identifying stakeholders, analyzing the interaction, sharing experience, and providing solutions (28). Other studies implied that value co-creation is divided into three stages, which later evolved into four stages: value consensus, value co-creation, value sharing, and value win-win (29). Interaction and resource integration are two key components of value co-creation. According to SD logic, value co-creation is generated through dialogue and participation with stakeholders in the value network. Service providers and customers play a role in co-creating value through their interactions (30). For example, the elderly are actively participating in high-quality SSC through frequent interaction and information exchange with other participants. This interaction refers to the integration of resources and capabilities by diverse participants to achieve value co-creation (31). Further, the efficiency of achieving co-creation may be increased when resources are integrated into the service process (32).

Currently, theoretical research lags behind practical innovation. Previous studies have focused more on how to increase the participation of the elderly in conventional care. Besides that, the research on SSC is still in initial stages. Little relevant research has touched on how smart senior care can achieve value co-creation. The theoretical framework for this research is the “Consensus-Co-creation-Sharing-Win-win” process, based on the above literature review. The purpose of this study is to investigate the driving factors and achievement paths for SSC to achieve value co-creation and then to establish a practical logic model for SSC value co-creation.

## Materials and methodology

This study utilizes a combination of multiple case studies and grounded theory. Since there is a lack of research on SSC value co-creation, these two research methods are feasible and beneficial when the problem is approached from a unique perspective. Second, this research focuses on the process of value co-creation in which multiple actors are engaged, as well as the construction of a practice logical framework for SSC value co-creation. Data collection and analysis, data coding, and model generation are the three parts of the methodological framework, and the script data is coded and summarized sentence by sentence (33). Continuous comparison, analysis, induction, and generalization were used to investigate the logical connections between the SSC concepts and categories in the literary sources. After theoretical saturation, research obtains a new logical model.

### Sampling

This study uses “Typical Cases of Chinese Elderly Service Industry Development” as research materials. These materials are 75 typical cases of senior care services selected by the National Development and Reform Commission, the Ministry of Civil Affairs and the National Office of Aging, after local submission, assessment, review, and online publication in 2017. This collection is produced together by a combination of government departments to highlight and promote the benefits of Chinese elderly service markets in recent years. It has diagnostic, demonstration, and promotional effects on the elderly service industry. Therefore, such materials have the characteristics of authority, authenticity, and reliability and are suitable to be selected as the script materials for this study. In this compilation, all the cases related to the theme of SSC (10 cases in total) are selected in Table 1. Simultaneously, each case content is supplemented by the department's official website and internet news, assembling a total of 53,000 words of analysis script.

**Table 1:** Source type and text statistics.

**No**.	**Case**	**Location**	**Typical cases (thousand words)**	**Network information (thousand words)**
A	Tongxiang City Wuzhen Internet plus Senior Care Service model	Eastern	3.823	2.327
B	Luoyang City Home Community SSC Innovation Model	Central	4.194	3.606
C	Ningxia Internet plus Senior Care Service Model	Northwestern	3.766	1.763
D	Hangzhou City SSC Service New Model	Eastern	2.764	1.368
E	Maanshan City Internet plus Senior Care Service Model	Eastern	2.45	0.881
F	Wuzhou City “cloud family” Internet plus community home care service	Southern	3.454	0.976
G	Case of senior care services in mountainous areas of Datian County	Eastern	2.871	2.355
H	Changzhou City Healthy Aging Service Industry Cluster Area	Eastern	4.082	2.781
I	Yantai City “Medical, Nursing, and Rehabilitation” Integrated Demonstration Plot	Eastern	3.558	1.978
J	Case of Shanxi Province Ruiquan Senior Care Service Co.	Northwestern	3.483	1.124

### Research process

The process of this study included data import, coding, data analysis, and model construction based on grounded theory. Three levels of coding are used to summarize the raw data, including open coding, axial coding, and selective coding. Coding and analysis can be performed simultaneously (34). Theoretical saturation is followed during coding. This means that scripted data have reached theoretical saturation when they can no longer be extended to new ideas or categories. This signifies that sample coding has ceased, and additional analysis and model creation has begun.

### Open coding

The SSC content is extracted from the case by sentence-by-sentence analysis. The final coding will include two parts: case number and sentence number. For example, the supplementary material for Tongxiang City's new model of senior care service is numbered “As”, and the third sentence in the case is numbered “As-3”. Based on understanding the text content, the statements are conceptualized. The initial concepts are then derived by removing duplicate items, combining synonyms, and classifying them. Eventually, in this section, 32 categories are identified. Due to the limitation of space, only representative codes are listed in this paper, as shown in Table 2.

**Table 2:** Open coding example.

**Concepts**	**Concept codes**	**Reference Points**	**Categories**
Insisting on the “combination of medical care, education and health” model to fill the gap in the semi-self-care and non-self-care elderly market (F-24)	Disabled Elderly	11	Service object
The municipal government attaches great importance to the development of elderly services (C-2)	Government Attention	6	Government support
The municipal government provides 150,000 yuan per year for home care service centers and 300,000 to 500,000 yuan from the welfare fund (C-4)	Financial Support	11	Economic support
Installing intelligent care equipment for eligible older people (A-13)	Intelligent Care Equipment	21	Technology application
ECG testing of elderly patients with sudden illness using Internet remote lead technology (Gs-14)	Emergency Assistance	24	Service delivery
The school's Retirement Office, Bureau of Civil Affairs, and service agency have reached a three-way cooperation (Js-4)	Cooperation Intention	39	Multi-actor collaboration
With expanding social influence and growing brand strength, Ruiquan Senior Care has become one of the most successful organizations (J-19)	Brand Influence	11	Demonstration effect
The platform service operating company develops “personalized and precise service” solutions (D-8)	Personalized Services	9	Personalized elements
The service provider offers discounted rates to all seniors at their own expense (E-13)	Low-cost Services	5	Special price
Effective integration of resources by combining medical and nursing care (B-13)	Integrated Services	17	Resource integration
Dozens of older people come to gather every day and live a happy and warm life (Hs-4)	Happy Life	8	Pleasant mood

### Axial coding

Axial coding serves as a bridge between empirical description and conceptual analysis. In other words, this part refines and separates the categories obtained from open coding, then examines connections and potential logical relationships among the categories, and finally extracts the main categories that govern the others. Five main categories and 17 sub-categories are extracted and defined in this paper (Table 3). Table 4 shows the results of the main coding.

**Table 3:** Axial coding.

**Main categories**	**Sub-categories**	**Categories**
A1 Multi-actor value consensus	B1 Need Identification	C1 Service Object C2 Service Subject
A2 Co-creation environment establishment	B2 Institutional Guarantee B3 Market Empowerment B4 Technical Intake B5 Emotional Support B6 Service Interaction B7 Reciprocity Norm	C3 Government Support C4 Policy Making C5 Economic Support C6 Non-government Investment C7 Industry Development C8 Technology Application C9 Intelligent Platform C10 Data Operation C11 Life Care C12 Interactive Exchange C13 Service Delivery C14 Multi-actor Collaboration C15 Demonstration Effect C16 Resource Emergence
A3 Practical value co-creation	B8 Functional Value B9 Economic Value B10 Social Value	C17 Personalized Elements C18 Inelastic Demand C19 Social Relationship Building C20 Group Identity C21 Rewards C22 Special Price
A4 Public value sharing	B11 Service Quality B12 Capacity Excavation B13 Resident Participation B14 Service Supply Innovation	C23 Service Evaluation C24 Service Regulation C25 Capacity Enhancement C26 Knowledge Value C27 Active Participation C28 Conception Renew C29 Resource Integration
A5 Diffusion of service added value	B15 Cultural Output B16 Hedonistic Value B17 Technology Collaboration	C30 Ecological Culture C31 Pleasant Mood C32 Technology Empowerment

**Table 4:** Definition of sub-category.

**Sub-category**	**Definition**
Need identification	Stakeholders' various needs for SSC services
Institutional guarantee	The government encourages multi-body participation through document development and policy support
Technical intake	Provide senior care services utilizing intelligent technology
Market empowerment	To meet the needs of the elderly as the primary goal in the market competition, give full play to the role of market mechanisms
Emotional support	The elderly get emotional care through interaction
Service interaction	Formation of collaborative mechanism among multiple actors to jointly promote the development of the SSC industry
Reciprocity norm	Equal trust between participants and gradually form a standardized service order
Functional value	The value that the elder feels about SSC services and product performance
Economic value	Economic benefits from participating in interactions
Social value	Develop social relationships or foster a sense of group belonging by participating in interactions
Service quality	Service satisfaction evaluation and service supervision through feedback
Capacity excavation	Personal improvement through participation in interactions
Resident participation	Enhancing the initiative of the elderly to participate is an essential condition for realizing value co-creation
Service supply innovation	Including concept renewal and technological change
Hedonistic value	The pleasure of participating in interactions
Cultural output	The elderly have a high sense of identification with SSCs, which produces a willingness to co-create value
Technology collaboration	Key technologies and resource introductions are integrated with service capabilities to reconstruct digital resource capabilities

### Selective coding

**Figure 1:**
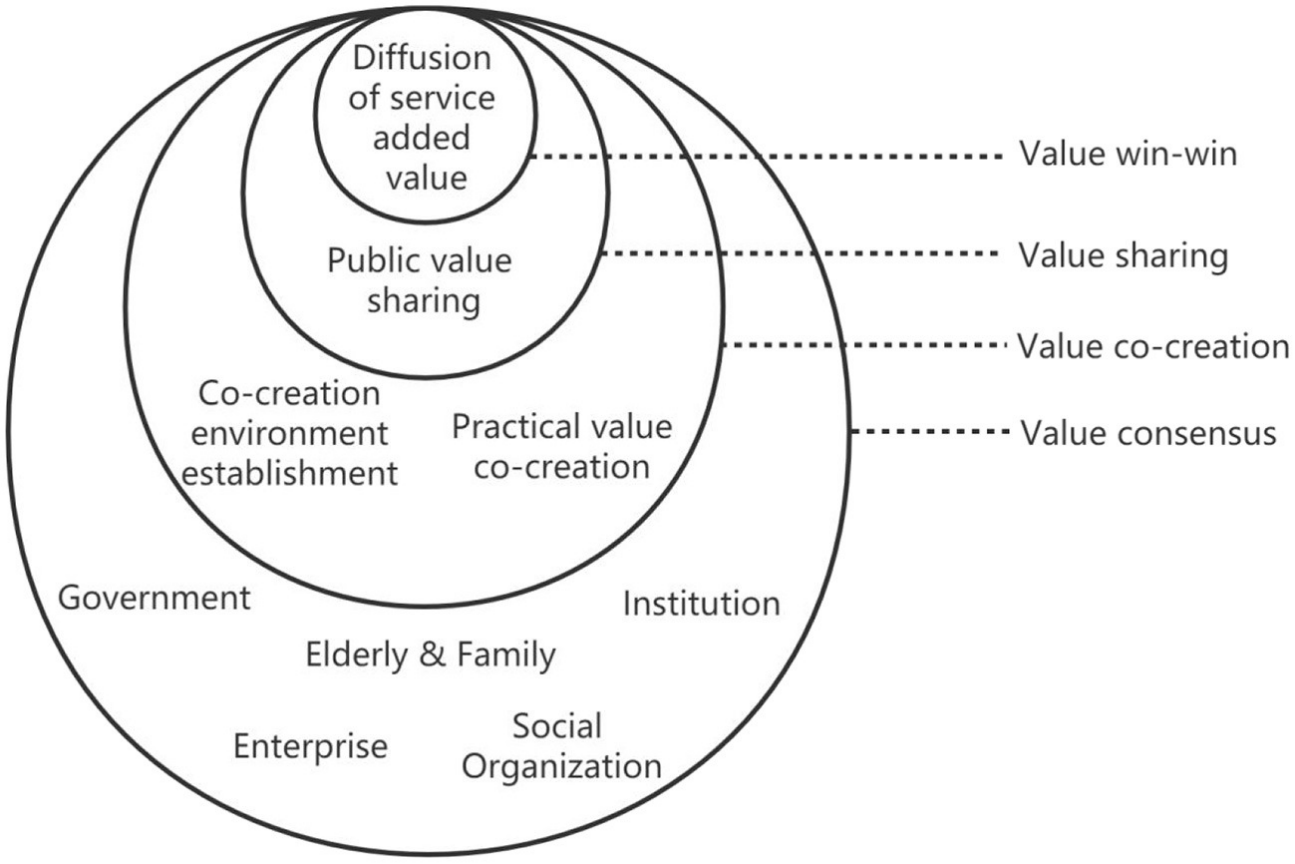
Conceptual model of SSC value co-creation.

Selective coding entails selecting core categories and relating them to others in a systematic way. The interrelationships are then verified, and the separate concepts are reassembled in the form of a “storyline” (21). The plot of the narrative is as follows: the Chinese SSC model is based on the interaction of multiple actors to achieve value consensus, and the interaction of actors exists in all aspects of the practice and has an impact on it; “co-creation environment establishment” and “practical value co-creation” are the causal conditions of value co-creation; “public value sharing” is the action strategy that constitutes the behavior and phenomenon of value sharing and finally forms the win-win result of “service added value diffusion”. Accordingly, the core scope of selective coding can express as a conceptual model of SSC value co-creation through a four-stage process of “value consensus-value co-creation-value sharing-value win-win” as shown in Figure 1.

### Saturation test

The saturation test is conducted to check the theoretical saturation of the coding. The remaining two examples have been coded, conceptualized, and categorized. If no new concepts emerge, so the script materials have been thoroughly explored, and the theory has reached a reasonable saturation level.

## Result

### Value consensus

SSC services involve a wide range of actors. Specifically, the SSC value co-creation network is formed by different participants, such as government, social organizations, enterprises, institutions, the elderly, and family members, all of whom have different demands and behavioral patterns (2).

“Ma City Civil Affairs Bureau staff: the city's civil affairs department will encourage all kinds of stakeholders to participate in SSC in order to promote the participation of multiple actors.” (Gs, Location 12)

Needs identification is the initial stage in value co-creation (35). That means all stakeholders search for needs and resources and reach a consensus on values (36). The community mainly represents the government position. Communities need to use third-party forces or innovative models to solve problems when faced with insufficient resources to accomplish governance tasks. At the same time, communities expect to help residents to raise awareness of SSC services.

“... integrate specialized service teams and volunteer organizations to provide three types of services to different elderly customs: public service, low-paid service, and paid service.” (D, Location 14)

Social organizations actively cooperate with stakeholders who can provide lower-cost SSC to absorb their service, technology, and talent resources to realize their interests and public interests.

“The Healthtop company is responsible for the project's operation, specifically customizing the five service packages of life care, community culture, catering, health management, and professional care.” (A, Location 12)

Enterprises achieve the integration of community services and heterogeneous resources, both public and commercial services, through intelligent online platforms (37).

“In order to achieve full institutional coverage, SSC service centers are established in county towns, and home-based SSC service stations are established in townships.” (H, Location 13)

Medical institutions or care institutions receive support and guidance from the government and social organizations to provide higher-quality services (38). Such services are provided to the elderly with disabilities, living alone, chronic diseases, and poverty.

“Institutions such as Senior Apartments, Community Care Centers, Senior Activity Centers, and Nursing Homes have actively integrated their resources with the SSC with the support of the county government and the Civil Affairs Bureau. To improve the effectiveness of the SSC, multiple actors have fully used existing social resources and organized a group of volunteers.” (H, Location 29)

SSC covers the government, institutions, social organizations, enterprises, the elderly, and their families. Adaptability and initiative are two characteristics of such actors. Based on following the value consensus, multiple actors exchange systems, funding, people, and technology through interactions and resource sharing without much prompting. When other components or the environment change, adaptive individuals may adjust their structure and behavior through experience and learning (39). The value consensus stage, where resources are separately identified, constitutes the initial stage of value co-creation.

### Value co-creation

The environmental establishment and practical value of SSC are examined in depth in this research. The co-creation environment attracts multiple actors to participate in SSC value co-creation through institutional guarantee, technical intake, market empowerment, emotional support, service interaction, and reciprocity norm. Following that, the practical value represents the physiological, safety, and emotional satisfaction users obtain through SSC online and offline integration, including social value, economic value, and functional value. This study considers practical value as a low level of needs value and the primary extrinsic motivation for the elderly to participate in value co-creation.

#### Co-creation environment establishment

##### Institutional guarantee

“Government Clarifies the leading role of SSC.” (I, Location 8)

“The city government builds an SSC service model with features of Luoyang City.” (C, Location 2)

“The government formulated and introduced Implementation Plan for SSC Activities in Datian County.” (H, Location 10)

In SSC, the institutional guarantee is critical. Institutional design and policy formulation by the government are prerequisites for achieving value co-creation. First, the government clarifies the leading position of SSC and assists with service and product optimization. Second, the government regulates the SSC service process and controls service quality by formulating policies to form deep interactions and reciprocal norms among multiple actors eventually (40).

##### Technical intake

“Using Big Data, Internet of Things, Artificial Intelligence and other technologies.” (E, Location 4)

AI, big data, and other modern technologies have lowered the “barriers” to participation in SSC for the elderly. The interaction between government and members has become more convenient and efficient. As a result, technology intake may provide opportunities and environments for society members to engage in co-creation activities, enrich co-creation content, and optimize co-creation processes.

##### Market empowerment

“To support private capital and social forces to enter the field of elderly services.” (E, Position 4)

In the SSC value co-creation scenario, the government stimulates local enterprises, social institutions, and other private capital to participate in the design and improvement of the SSC project through a market-based approach. In terms of market empowerment, the market mechanism emphasizes “the survival of the fittest”. Enterprises should prioritize satisfying the demands of the elderly, enhance the accessibility of SSC services, and provide high-quality services for the elderly in the market competition.

##### Emotional support

“Children of the elderly can remotely check their parents' condition in real-time through a mobile app and book relevant SSC services for their parents.” (I, position 27)

Currently, digital literacy among the elderly is relatively insufficient. The elderly are encouraged to engage actively in SSC *via* family intergenerational communication and digital feedback. Meanwhile, intergenerational support is a powerful technique to increase family support for SSC and a social buffer mechanism to alleviate mental isolation (41).

##### Service interaction

“The system is used online for the collection and distribution of service requests, and the APP is used offline for the positioning and supervision of services.” (Bs, position 15)

Each value co-creator establishes a connection by exchanging interests and requests. Compared with the traditional senior care service, SSC combines online and offline, which better activates the central position of value co-creation of the elderly (42). Demanders and providers of senior care services might well be efficiently matched utilizing the SSC service information platform in the service interaction. To increase the convenience and timeliness of senior care services, the platform can connect demand information to service providers in real-time.

##### Reciprocity norm

“Encourage and support the development of new forms of services, and cultivate several leading enterprises with strong power and high-profile service brands.” (E, position 27)

The actors' reciprocity norm efficiently promotes the SSC industry's innovative development and creates public value (43). Meanwhile, the government implements financial incentives, honorary awards, and preferred experiences to increase enthusiasm for enterprise and consumer engagement, encouraging social members to participate and interact in-depth to achieve social value consensus. After that, the social impact of value co-creation forms public value and cultural output and paves the way for the next stage of value co-creation.

#### Practical value co-creation

“SSC services are expanded to include personalized service programs that are urgently needed in the lives of seniors.” (Ds, position 9)

“...conducted more than 15,000 times of various services, with 99.5% service satisfaction on return visits.” (E, Location 32)

“Service providers integrate regional service resources, and seniors can enjoy services for free or at low cost.” (E, Location 14)

SSC meets the individual needs and rigid demands of senior health through personalized services. The comfort, simplicity of use, and humanization of the service represent its functional value (44). Furthermore, the services may become more convenient and easy to use, which will enhance the functional value acquired by customers. Social value is the development or cultivation of group belonging through participation and interaction. Interaction improves group cohesion and satisfaction with the service, boosting group identification and belonging. Economic value refers to the economic benefits that the elderly obtain by engaging in interaction. Economic benefits include services at favorable prices, gifts, or virtual community rewards to meet their actual needs. Moreover, economic value has a beneficial impact on the appraisal of services and brands, resulting in a greater reliance on and liking for them (45).

### Value sharing

“The SSC Center is a combination of SSC platform, restaurant, club activities, dance, painting and calligraphy, health management and other functions.” (As, location 16)

“The SSC Center provides leisure services for seniors such as swimming, fishing, vegetable gardening, and flower raising, and produces and supplies pollution-free seafood and vegetables.” (F, Location 26)

Public value sharing brings positive and extensive impacts, such as capacity excavation, service supply innovation, service quality improvement, and wide resident participation. The whole sharing process is driven by the government, with the involvement of companies, institutions, the elderly, and families to establish a service ecosystem (19). The deep interaction and resource integration of all elements within its system jointly create public values (46). This promotes the innovation and optimization of SSC products or services and ultimately creates a process of public value sharing. SSC innovates service forms, optimizes service experience, and constantly improves service quality in the public value-sharing cycle. Public value sharing may draw the attention and involvement of more people and provide support for the next stage of value co-creation.

### Value win-win

“...meet the diversified needs of the elderly, keep them happy and healthy, and enjoy their senior life happily.” (F, Location 16)

“...create an SSC industrial chain integrating research, training, production, sales and service.” (F, Location 27)

In the process of value co-creation of SSC cooperative supply, the service added value is the part that is higher than the value of SSC service itself created by the participation of multiple actors. In the era of big data, digital empowerment has dramatically increased the added value of SSC service value co-creation. The integration of multiple actors makes SSC supply and demand match accurately and improves the efficiency of service operation, and promotes the technology collaboration among multiple actors (47). SSC services may meet the hedonistic need of the elderly from their heart. Because spiritual value originates from the customer's intrinsic motivation, it is a purely psychological and spiritual need. SSC not only develops intelligent service equipment but also pays attention to the actual needs of the elderly from physical to spiritual levels (48).

Cultural factors are the key to the participation of multiple actors in value co-creation. There are two reasons for this: first, the elderly have a high sense of identification with the culture of senior care. Secondly, the cultural output provided by SSC is the driving force for the elderly to change from “passive consumption” to “active co-creation” (20). In other words, when deep interaction and cultural connotation match, the willingness to participate in value co-creation will be generated (49).

In the stage of value win-win, the government, enterprises, institutions, social organizations, and the elderly have achieved the intersection and integration. The resources of SSC have been effectively utilized, transformed, and fed back, which constitutes the final stage of value co-creation.

### Practical logic model of SSC value co-creation

SSC value co-creation is a dynamic evolutionary and continuous optimization process. In this study, institutional guarantee, technical intake, market empowerment, emotional support, service interaction, and reciprocity norm are essential driving factors of SSC value co-creation. The behavioral processes of SSC value co-creation include multi-actor value consensus, co-creation environment establishment, practical value co-creation, public value sharing, and diffusion of service added value.

**Figure 2:**
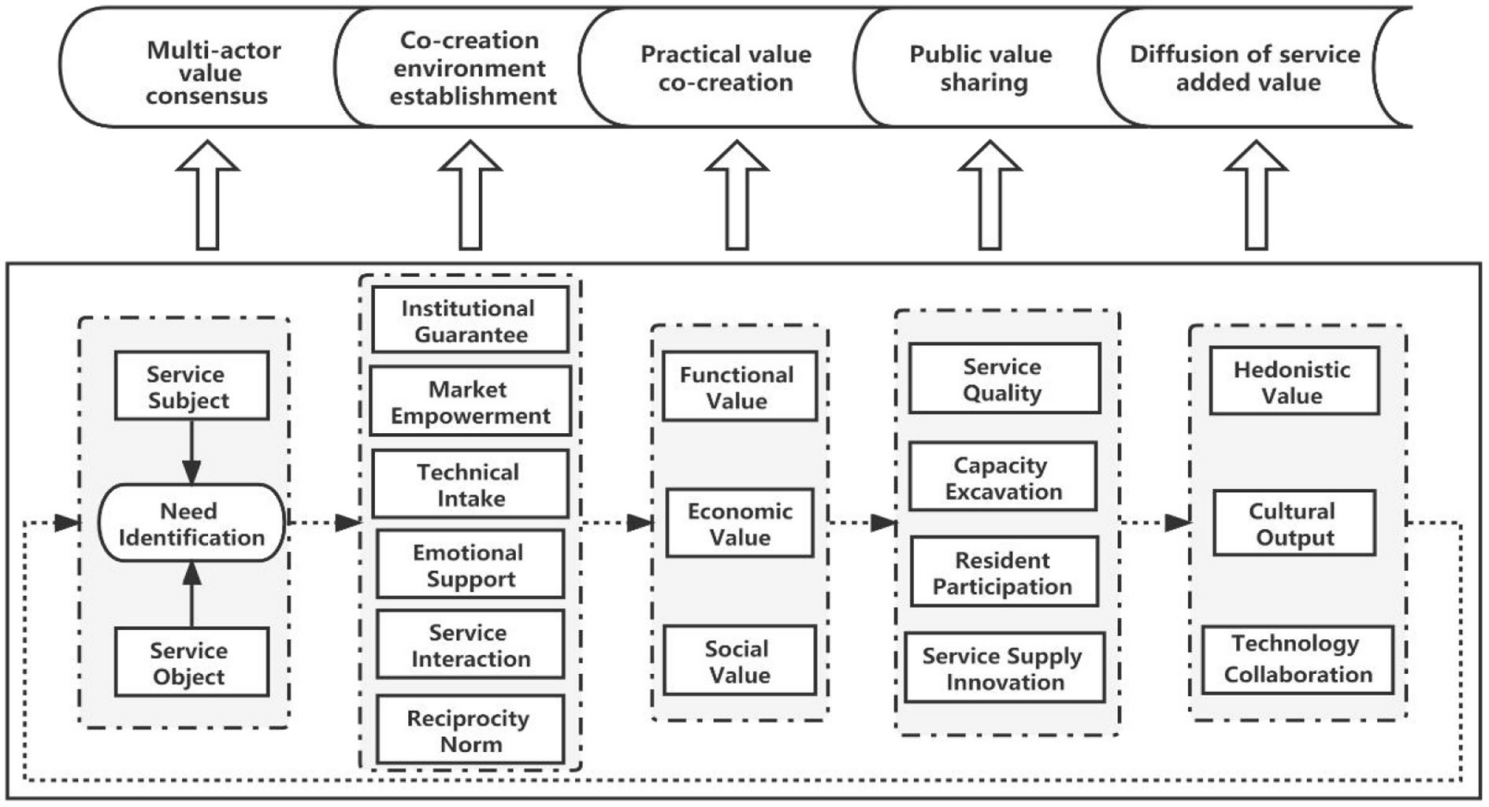
Practical logic model of SSC value co-creation.

A practical logic model of multiple actors' co-creation is established in this research. Figure 2 is gradually formed under the role of driving factors and behavior processes. The interaction of its internal elements will influence the behavioral process and results of value co-creation of SSC services. The value co-creation network, which includes government, enterprises, institutions, social organizations, and the elderly, is sociable. In addition, the action result of the Practical logic model is value co-creation, which may be seen as a mediating factor that impacts the whole value co-creation process (50). The internal interaction and resource integration of the Practical logic model drives the elderly and families as service consumers to form a dynamic, balanced, and interactive system with government, enterprises, and other elements. Participants use their knowledge, skills, and experience to contribute to value creation (51).

## Discussion

The value co-creation theory under the SD Logic provides guidelines for government public affairs governance and public service supply and encourages social forces to participate in public service value co-creation. While exploring the theoretical aspects of SSC value co-creation, this research also proposes a few points for discussion on the construction and development of public services in SSC.

The critical issues for the government are how to establish a co-creation environment for effective communication, active participation, and continuous interaction among members of society. As a result, it is required to unite social members' awareness and carry out various value co-creation under a unified value consensus dispatch. The government should set up an easy-to-accept method of service delivery for the elderly, as well as a more open and equal co-creation environment. This has the potential to provide hedonistic value and also cultural output. At the same time, it may help SSC service enterprises and institutions enhance their brand image and public service goods (52).

When enterprises and institutions participate in SSC services delivery, they consider things such as upfront construction costs, development profitability, and capital recovery period. This influences their decision to give services to elderly adults in more developed locations. Alternatively, they choose to relocate to locations where the government provides significant incentives, supportive policies, and a favorable investment environment (53). In this research, the vast majority of the instances are in such places. This is due to the fact that in more open markets, the elderly's high economic paying capacity and diverse SSC demands ensure long-term profitability. Market actors are active in the SSC service market and engage in all elements of senior care services because of the market environment under the double guarantee. On the contrary, for regions with lower economic development levels, especially rural areas, low consumption demand and traditional cultural concepts severely restrict the choice of SSC services for the elderly. As a result, the rural SSC market lacks the necessary growth velocity to attract active engagement from social forces, leading to a failure to exploit the function of fundamental public service equalization fully. To improve the positive impact of value co-creation, it is vital to raise visibility *via* social marketing in the face of such issues. This is to encourage more social actors to pay attention to and participate in the design of SSC service products, service quality control, and service optimization. A new round of value co-creation can be formed so that the SSC service level can be continuously improved and enhanced.

The paucity of current senior care resources in China significantly impacts the elderly's perception. With IoTs and big data as the foundational technological support, SSC combines elder care service resources and distributes them logically. Using the Internet or new media platforms in value co-creation increases the public awareness of participating in public services (36). Actively building an online interactive platform with a digital service platform as an important carrier can broaden the communication channels with multiple actors. As a result, it achieves the modernization of facilities, as well as the convenience and quality of SSC services, and becomes a new option for addressing the shortfall of total senior care resource supply. China should advocate the SSC model, which applies modern technology to many areas of need for the elderly.

## Conclusion

This paper adopts a multi-case study approach and selects the value co-creation process of multiple actors in SSC services as the research object. The study effectively identifies the process of interaction between actors and the process of resource integration by grounded theory. The study constructs a practical logic model of the value co-creation process, which corresponds to the actions and results of value consensus, value co-creation, value sharing, and value win-win. This study confirms that SSC service providers should be oriented to the needs of the elderly and ultimately achieve value co-creation. Different actors participate in SSC and provide differentiated and integrated services for the elderly. Our results provide evidence of how value co-creation is achieved in SSC services, revealing its driving factors and behavioral processes. This way can effectively solve the current problem of insufficient supply capacity and limited resources of senior care services and alleviate the gap of equalization of public services.

The innovation of this paper is mainly reflected in the following three aspects. First, the concept of SSC value co-creation is defined. The driving factors of SSC value co-creation are identified, including institutional guarantee, technical intake, market empowerment, emotional support, service interaction, and reciprocity norm. Second, this study proposes that SSC service value co-creation is a dynamic evolutionary and continuous optimization process and identifies five behavioral processes, including multi-actor value consensus, co-creation environment establishment, practical value co-creation, public value sharing, and diffusion of service added value. Finally, many studies have discussed value co-creation in corporate services. However, there is a lack of in-depth analysis of the mechanism and model behind the phenomenon of SSC value co-creation. This study enriches the theory by combining SSC and value co-creation theory and refines a practical logic model of SSC value co-creation.

This study explores the participation of multiple actors in SSC value co-creation through the grounded theory method, but there are certain shortcomings. First, as a qualitative research method, the grounded theory is analyzed according to the subjective judgment of the researcher, and there may be a particular bias in the research process. Secondly, the cases analyzed in this study are typical case inferences. In the future, further expansion of the case sample is needed to deepen the study.

## Data availability statement

The original contributions presented in the study are included in the article/supplementary material, further inquiries can be directed to the corresponding author.

## Author contributions

TP and JH: materials and methodology. TP and JY: coding. TP: validation, resources, and writing—review and editing. LF and TP: result analysis. LF: writing—original draft preparation, visualization, supervision, project administration, and funding acquisition. All authors have read and agreed to the published version of the manuscript.

## Funding

This research was funded by the National Social Science Foundation of China, grant number 20AGL034.

## Conflict of interest

The authors declare that the research was conducted in the absence of any commercial or financial relationships that could be construed as a potential conflict of interest.

## Publisher's note

All claims expressed in this article are solely those of the authors and do not necessarily represent those of their affiliated organizations, or those of the publisher, the editors and the reviewers. Any product that may be evaluated in this article, or claim that may be made by its manufacturer, is not guaranteed or endorsed by the publisher.
